# Comparison of the Binding of Reversible Inhibitors to Human Butyrylcholinesterase and Acetylcholinesterase: A Crystallographic, Kinetic and Calorimetric Study

**DOI:** 10.3390/molecules22122098

**Published:** 2017-11-29

**Authors:** Terrone L. Rosenberry, Xavier Brazzolotto, Ian R. Macdonald, Marielle Wandhammer, Marie Trovaslet-Leroy, Sultan Darvesh, Florian Nachon

**Affiliations:** 1 Departments of Neuroscience and Pharmacology, Mayo Clinic College of Medicine, Jacksonville, FL 32224, USA; rosenberry@mayo.edu; 2 Département de Toxicologie et Risques Chimiques, Institut de Recherche Biomédicale des Armées, 91220 Brétigny-sur-Orge, France; xavier.brazzolotto@chemdef.fr (X.B.); marielle.wandhammer@chemdef.fr (M.W.); marie.trovaslet-leroy@chemdef.fr (M.T.-L.); 3 Department of Diagnostic Radiology, Dalhousie University, Halifax, NS B3H 4R2, Canada; Ian.MacDonald@Dal.Ca; 4 Department of Medical Neuroscience, Dalhousie University, Halifax, NS B3H 4R2, Canada; Sultan.Darvesh@Dal.Ca; 5 Department of Chemistry, Mount Saint Vincent University, Halifax, NS B3M 2J6, Canada; 6 Department of Medicine (Neurology and Geriatric Medicine), Dalhousie University, Halifax, NS B3H 4R2, Canada

**Keywords:** acetylcholinesterase, butyrylcholinesterase, crystal structure, kinetics, isothermal titration calorimetry

## Abstract

Acetylcholinesterase (AChE) and butyrylcholinesterase (BChE) hydrolyze the neurotransmitter acetylcholine and, thereby, function as coregulators of cholinergic neurotransmission. Although closely related, these enzymes display very different substrate specificities that only partially overlap. This disparity is largely due to differences in the number of aromatic residues lining the active site gorge, which leads to large differences in the shape of the gorge and potentially to distinct interactions with an individual ligand. Considerable structural information is available for the binding of a wide diversity of ligands to AChE. In contrast, structural data on the binding of reversible ligands to BChE are lacking. In a recent effort, an inhibitor competition approach was used to probe the overlap of ligand binding sites in BChE. Here, we extend this study by solving the crystal structures of human BChE in complex with five reversible ligands, namely, decamethonium, thioflavin T, propidium, huprine, and ethopropazine. We compare these structures to equivalent AChE complexes when available in the protein data bank and supplement this comparison with kinetic data and observations from isothermal titration calorimetry. This new information now allows us to define the binding mode of various ligand families and will be of importance in designing specific reversible ligands of BChE that behave as inhibitors or reactivators.

## 1. Introduction

**Figure 1:**
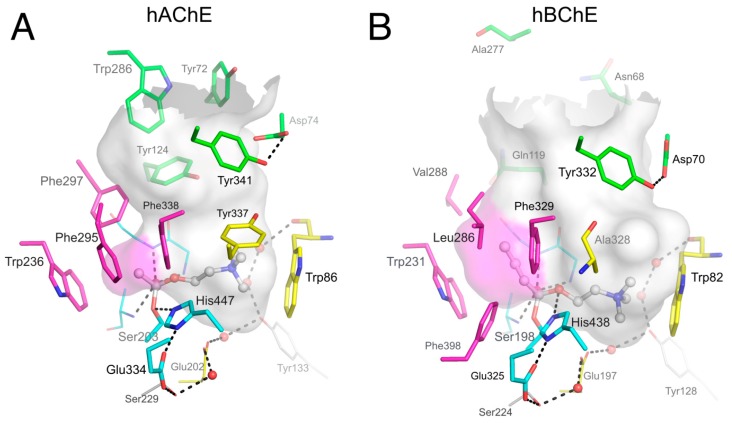
Active site gorges of human acetylcholinesterase (hAChE) (**A**; pdb 4ey4) and human butyrylcholinesterase (hBChE) (**B**; pdb 1p0i). The acylation transition state of a substrate molecule of acetylcholine (ACh) or butyrylcholine (BCh) is modelled and represented in ball and stick. The gorge is depicted by its molecular surface (semi-transparent gray and magenta for the acyl-binding pocket). The main residues are represented. The catalytic triad (in sticks) and oxyanion hole residues (in lines) of the A-site are in cyan. The acyl-binding pocket of both enzymes is in magenta. The key aromatic residues of the choline-binding pocket in the A-site are in yellow. P-site residues located at the rim of the gorge are in green. Conserved structural water molecules are represented in red spheres. The dense hydrogen bond network of the A-site is represented in dashed lines.

Acetylcholinesterase (AChE, EC 3.1.1.7) and butyrylcholinesterase (BChE, EC 3.1.1.8) are serine hydrolase enzymes that catalyze the hydrolysis of acetylcholine [1]. X-ray crystallography analysis of these cholinesterases [2,3] has established that catalysis takes place in a 20-Å deep active site gorge and involves a catalytic triad of serine, histidine, and glutamate residues located near the bottom of the gorge (Figure 1), denoted the acylation or A-site. The region near the rim of the gorge has been denoted the peripheral site or P-site.

The P-site in AChE is lined with aromatic residues that play a key role in the binding and orientation of aromatic and/or cationic substrates on their way from the P-site to the A-site. For example, aromatic residues Trp286, Tyr124, Tyr72 and Tyr341 in the human AChE (hAChE) P-site form π-cation interactions with acetylcholine and orient it to slide down to Trp86 and Tyr337 of the choline-binding pocket in the A-site, where it is correctly aligned with the catalytic serine [4,5]. When acetylcholine is correctly oriented for catalysis, Phe295, Phe297, and Trp236 form an acyl-binding pocket that tightly accommodates the acetyl part of the substrate. At high substrate levels, the activity of AChE decreases [6,7], a phenomenon denoted substrate inhibition, that is thought to occur through steric blockade of product release that results from the binding of an additional substrate molecule to the P-site [8]. The active site gorge of AChE has been mapped in detail by AChE mutant studies [9,10] as well as by X-ray crystallography of the enzyme bound to ligands that interact with various regions of this gorge [11,12,13,14]. Prototypical P-site ligands like propidium and thioflavin T (ThT) bind to the P-site of AChE, while the A-site ligand edrophonium binds to the choline binding site, thus interfering with substrate access to the A-site. X-ray crystallography studies corroborated a kinetic approach that employed binding site competition between these inhibitors to help define locations of ligand binding in the AChE active site gorge [15].

Early comparison of AChE and BChE showed that most differences in ligand binding specificity between the two enzymes arise from differences in the number of aromatic residues in the gorge [9,16]. Among the 10 aromatic residues interacting with ligands in the hAChE gorge, only 4 remain in the human BChE (hBChE) gorge: Tyr332 (Tyr341 in AChE) in the P-site; and Trp82, Phe329 and Trp231 (Trp86, Phe338 and Trp236 in AChE) in the A-site (Figure 1). A direct consequence of these differences is that, at concentrations where substrate inhibition is present in AChE, substrate activation is observed in BChE. This activation is mediated by the binding of a second substrate molecule to the gorge in either the enzyme-substrate complex or the acylated BChE enzyme [2], to accelerate catalysis by stabilizing intermediates [17]. Such substrate activation involving the enzyme-substrate complex has also been observed for certain substrates with AChE [18]. The residue Tyr332 has been implicated in the binding of substrates to BChE, suggesting that this amino acid residue is part of a P-site in this enzyme [19,20,21]. Anionic Asp70 (Asp74 in AChE) is H-bonded to Tyr332 and is the other essential residue of the BChE P-site. Asp70 and Trp82 both belong to the cysteine Ω–loop, thus establishing a direct connection between the P-site and the choline-binding pocket in the A-site. Mutation of Asp70 in BChE, but also of Asp74 in AChE, to an uncharged glycine residue largely eliminates substrate activation in BChE and substrate inhibition of AChE [7,22].

Since inhibitor binding site competition analysis and mutant studies were successful in mapping ligand binding to the AChE P-site, a similar approach was made to probe the BChE active site gorge [23]. Wild-type and mutant BChE species and the enzyme inhibitors ThT, propidium, edrophonium and two synthetic phenothiazine derivatives were examined, and the results indicated the participation of aryl residues (Phe329 and Tyr332) in the alpha helix (E-helix; residues 326–332) bordering the BChE active site gorge, along with the anionic aspartate residue (Asp70), in the binding of ligands to the P-site of the enzyme.

**Figure 2:**
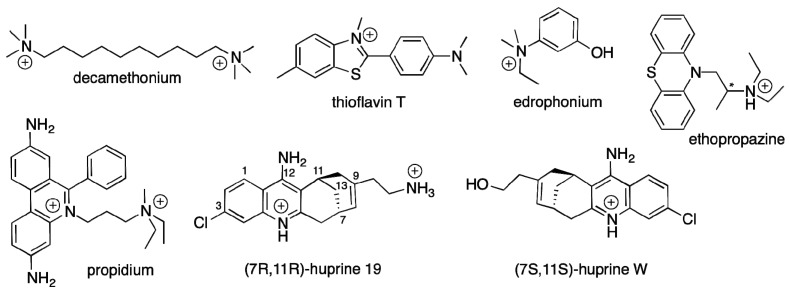
Chemical structures of the compounds used in this study. * denotes a chiral center.

Nevertheless, a lack of X-ray structures for a large diversity of reversible ligands bound to BChE still prevents a complete mapping of the BChE active site gorge. So far, structures with a few ligands are available. These include tacrine [24]; bis-aromatic pyridinium compounds [25], ZINC8924195, a nitroxoline derivative [26], dihydroindenylpiperidine naphthamide and benzylpiperidine naphthalene sulphonamide derivatives [27,28,29], and a carbazole derivative [30]. In the present study, we extend the kinetic study of Macdonald et al. with X-ray structures of BChE in complex with decamethonium, ThT, propidium, huprine 19, and ethopropazine, with kinetic data involving decamethonium and ThT, and with isothermal titration calorimetry (ITC) measurements. The chemical structures of the cholinesterase ligands used in the present study are shown in Figure 2.

## 2. Results

### 2.1. X-ray Structures of Human BChE-Ligand Complexes

#### 2.1.1. Decamethonium

**Figure 3:**
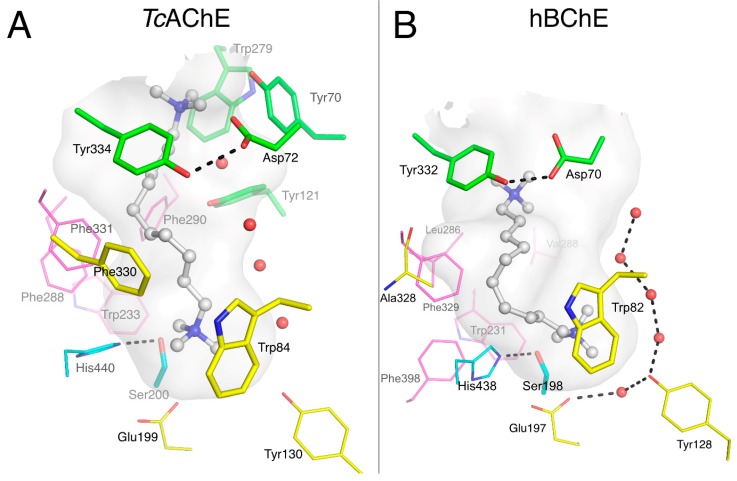
Complexes of decamethonium with *Tc*AChE (**A**; pdb 1acl) and hBChE (**B**; pdb 6ep4). The gorge is depicted by its molecular surface (semi-transparent gray). Nitrogen atoms are in blue and oxygen atoms in red. The ligand is represented in ball and stick format with carbon atoms in white. The residues in the vicinity of the ligand are represented in stick or lines. In the A-site, catalytic residues are in cyan, choline-binding pocket residues in yellow and acyl-binding pocket residues in magenta. P-site residues are in green. Conserved structural water molecules are represented in red spheres. The hydrogen bonds are represented in dashed lines.

Decamethonium is a prototypical dual binding site ligand of AChE that spans the active site gorge from the P-site to the choline binding pocket in the A-site, where it is stabilized by the cation-π interactions illustrated in its complex with *Torpedo californica* AChE (*Tc*AChE) [11] as shown in Figure 3A. One quaternary group interacts with Trp84 near the bottom of the gorge (3.7 Å), and the other interacts with a cluster of P-site residues, mainly Trp279 (3.2 Å), Tyr70 (3.2 Å), and Tyr121 (4.1Å). Phe330 lies parallel to the gorge surface, nicely accommodating the alkyl chain (3.3 Å). On a side note, *Tc*AChE is highly homologous to hAChE (58.5% identity) and shares identical active site residues, except for Tyr337 in hAChE substituted by Phe330 in *Tc*AChE. It follows that X-ray structures of *Tc*AChE-ligand complexes are generally identical to the complexes obtained in hAChE.

Crystals of the BChE_CHO_-decamethonium complex were grown by cocrystallization in the presence of 1 mM ligand. The structure was solved at 2.3 Å resolution. Decamethonium also spans the gorge of BChE from the gorge entrance to the choline binding pocket (Figure 3B), where the deeper quaternary group interacts with Trp82 (3.8 Å between C11 and indole plane) and Glu197-Oε1 (3.2 Å). It is noteworthy that this Glu197, which is conserved among cholinesterases, has an unusually high pKa and can be protonated at neutral pH [31]. It follows that the interaction of Glu197 with the quaternary ammonium moiety of decamethonium is not necessarily ionic but could involve a hydrogen bond [32]. The 10-carbon chain of the ligand closely follows the shape of the gorge surface toward Ala328, forms a 90° bend, and proceeds towards the gorge entrance where the second quaternary group is at the right distance to interact with Tyr332 at the P-site (3.7 Å between Tyr 332 aromatic centroid and C2 of decamethonium). The ability of the alkyl chain to fit in the void, otherwise filled by Phe330 in *Tc*AChE (replaced by Ala328 in hBChE), and to form a sharp bend, decreases the interatomic distance of the two quaternary nitrogens to 8.7 Å, compared to 11.7 Å in the *Tc*AChE complex. This structure provides further support that Tyr332 is a key element of the P-site of hBChE [20,21].

#### 2.1.2. Thioflavin T

**Figure 4:**
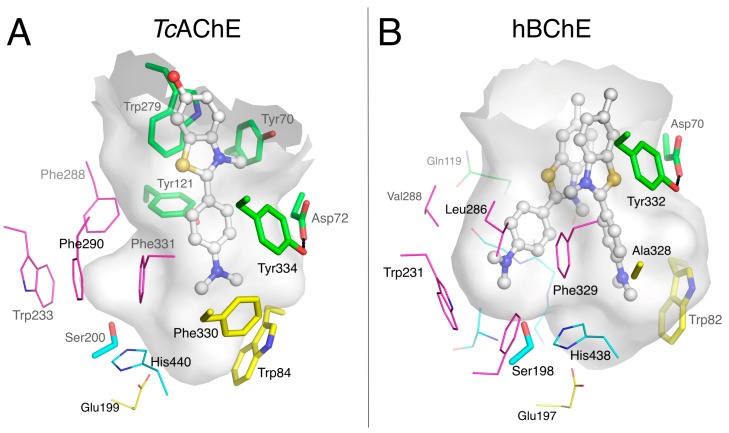
Complexes of thioflavin T with *Tc*AChE (**A**; pdb 2j3q) and hBChE (**B**; pdb 6esy). The gorge is depicted by its molecular surface (semi-transparent gray). Nitrogen atoms are in blue, oxygen atoms in red, sulfur atoms in yellow. The ligand is represented in ball and stick with carbon atoms in white. The residues in the vicinity of the ligand are represented in stick or lines. In the A-site, catalytic and oxyanion hole residues are in cyan, choline-binding pocket residues in yellow and acyl-binding pocket residues in magenta. P-site residues are in green.

Unlike decamethonium, ThT is not sufficiently long to span the P-site and the choline binding site pocket of *Tc*AChE [12]. The benzothiazole and dimethylaminophenyl rings, and the dimethylamino group of this ligand are coplanar and lay parallel to Trp279 and Tyr334 and Phe330, respectively (Figure 4A). The dimethylamino group is at 3.3 Å from the aromatic ring of Phe330 but remains far from the gorge bottom, at a distance of 8.5 Å from the carboxylate oxygens of Glu199. This position at the P-site allows concomitant binding of A-site ligands like edrophonium or m-(*N*,*N*,*N*-trimethylammonio)trifluoroacetophenone (TMTFA) through an adjustment of the orientation of Phe330 [12].

Crystals of the BChE_S2_-ThT complex were grown by cocrystallization in the presence of 0.5 mM ligand. The structure was solved at 2.8 Å resolution. The electron density visible in the gorge unambiguously revealed the presence of two molecules of ThT completely filling the wide active site gorge of hBChE (Figure 4B). The longitudinal axes of the two molecules formed an angle of 45° with each other. Their thiazole rings formed a remarkable aromatic stacking pile with Tyr332: 3.7-Å distance between the aromatic ring centroids of Tyr332 and the closest thiazole, and 3.9-Å distance between the thiazole centroids of the two ThT molecules. In absence of close counter anions, this stacking system likely helps in stabilizing the positive charge delocalized on each benzothiazole. The dimethylamino group of the molecule directly stacked against Tyr332 goes 3.1 Å deeper into the gorge compared to ThT in *Tc*AChE, at 6.4 Å from the carboxylate oxygens of Glu197, and therefore interacts directly with Trp82 of the choline binding pocket (3.8 Å of indole plane). The dimethylaminophenyl group of the second molecule fills the acyl-binding pocket with the dimethylamino group at 4.0 Å distance from Trp231 indole plane and one methyl group at 2.9 Å from Ser198-Oγ.

#### 2.1.3. Propidium

**Figure 5:**
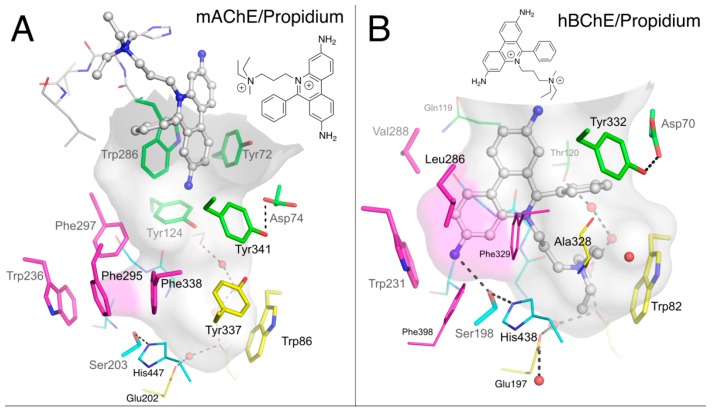

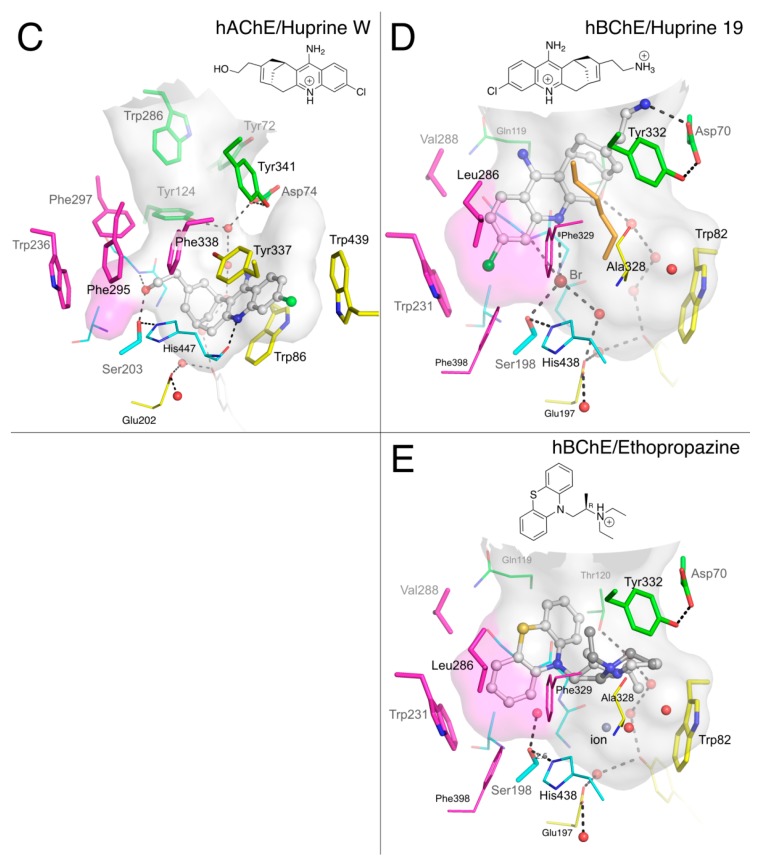
Complexes of propidium with mAChE (**A**; pdb 1n5r) and hBChE (**B**; pdb 6esj); huprine W with hAChE (**C**; pdb 4bdt); huprine 19 with hBChE (**D**; pdb 6eqq); and ethopropazine with hBChE (**E**; pdb 6eqp). The gorge is depicted by its molecular surface (semi-transparent gray and magenta for the acyl-binding pocket). Nitrogen atoms are in blue, oxygen in red, sulfur in yellow, chlorine in green, bromine in brown and other ion in grey. The ligand is represented in ball and stick format with carbon atoms in white. The residues in the vicinity of the ligand are represented in stick or lines. In the A-site, catalytic and oxyanion hole residues are in cyan, choline-binding pocket residues in yellow and acyl-binding pocket residues in magenta. P-site residues are in green. Water molecules are in red spheres. The hydrogen bonds are represented in dashed lines.

Propidium, which in AChE is unable to displace A-site ligands like edrophonium, greatly helped to define the P-site of AChE [33]. The crystal structure of the complex formed with mouse AChE (mAChE) confirmed that propidium binds at the gorge entrance, not overlapping with the A-site, with the phenanthridinium ring parallel-stacked with the indole ring of Trp286 and the extended alkyldiethylmethylammonium moiety lining the AChE molecular surface [14] (Figure 5A). It is noteworthy that, mAChE shares 89.2% identity with hAChE with strictly conserved active site gorge residues, and X-ray structures of the complexes are identical for the two enzymes.

Crystals of the BChE_S2_-propidium complex were grown by cocrystallization in the presence of 1 mM ligand. The structure of the complex was solved at 3.0 Å resolution. It is apparent, because of the absence of a residue equivalent to Trp286 in mAChE (or Trp279 in *Tc*AChE) at the gorge entrance, that propidium binds very differently to BChE and mostly fills the gorge (Figure 5B). The phenanthridinium ring is slotted into the groove of the acyl-binding pocket, with the amino group at interaction distance from Trp231 (2.8 Å to the center of the 6-carbon ring) and Ser198-Oγ (3.3 Å), and T-stacked to Phe329. The alkyldiethylmethylammonium moiety extends to Trp82 of the A-site at cation-π interaction distance (3.1 Å between the 6-carbon ring indole center and C5). The second ethyl group of the quaternary center docks against the propidium phenyl group (4 Å to aromatic plane), suggesting an intramolecular cation-π interaction. The propidium phenyl group is oriented toward the P-site residues Tyr332 and Asp70 (C16-CZ 3.6 Å distance and C15-Oε2 3.8 Å distance) providing additional, if not essential, opportunities for interaction.

#### 2.1.4. Huprines

Huprines constitute a family of nano- to femtomolar inhibitors of AChE that show specificity for A-site interaction [34]. The X-ray structures of various huprine derivatives with *Tc*AChE [35], mAChE [36], and hAChE [24] revealed that the 7S,11S isomers perfectly match the molecular surface of the choline-binding pocket in the A-site. The structure of a hydroxylated huprine derivative (Huprine W) bound to hAChE provides a fine illustration of the multiple interactions involved in the stabilization of the complex (Figure 5C). The main feature is the remarkable embedding of the chloroquinolinium moiety into an aromatic stacking pile involving Trp86/Huprine W/Tyr337/Phe338/Phe295/Trp236. Additional interactions include those of the polarized chlorine substituent with Trp439 (3.4 Å to indole plane) and of hydrogen bonds between the quinolinium nitrogen and the main chain carbonyl of His447 (2.8 Å) and between the hydroxyl group and the γ-hydroxyl of Ser203 (2.3 Å) and α-amine of Gly122 (2.9 Å).

We suggested previously that the three orders of magnitude decrease in inhibition activity of huprine W for BChE compared to that for AChE (IC_50_ = 1.2 µM vs. 1.1 nM) [37] was related to steric hindrance involving the chlorine substituent. This moiety does not fit readily into the BChE A-site and would likely force ligand binding at a different location in the gorge [24]. However, we failed to obtain crystals of the BChE huprine W complex that could have supported this hypothesis. Meanwhile, we showed that huprine 19, which differs from huprine W by the substitution of the primary hydroxyl by a primary amine and an opposite absolute configuration at C7 and C11 (Figure 2), showed an affinity similar to that of huprine W for BChE (IC_50_ = 0.9 µM vs. 1.2 µM) [37]. Huprine 19 generated an excellent Sepharose-based affinity resin that performed well in purifying BChE [38]. Thus, we made new attempts to obtain crystals of BChE_CHO_ in complex with huprine 19 by cocrystallization in the presence of 1 mM ligand and finally succeeded. The structure was solved at 2.4 Å resolution. As anticipated, the binding mode of huprine 19 to BChE differs dramatically from that of huprine W to AChE (Figure 5D). The electron density in the active site could be unambiguously assigned to the 7R/11R isomer of huprine 19, contrasting with the fact that the 7S/11S isomer of huprine W binds to AChE. The chloroquinolinium moiety is slotted into the acyl-binding pocket groove with the chlorine atom at interaction distance of Trp231 (3.4 Å to indole plane). This position of huprine 19 also is stabilized by the coincidental presence of a bromide counter-ion (large peak in the Fo-Fc map), which is H-bonded to the quinolinium nitrogen (3.7 Å), Ser198-Oγ (3.2 Å), the main chain nitrogens of Gly116 and Gly117 (3.4 and 3.6 Å), and a water molecule (2.9 Å) that itself is H-bonded to Glu197-Oε1 (2.8 Å). Such an anion located in the oxyanion hole is not unprecedented, as a fluoride ion was similarly positioned in a structure of the G117H mutant of BChE [39]. The primary ammonium of huprine 19 points toward the P-site, forming an H-bond to Asp70-Oδ1 (2.8 Å) and to Ser72-Oγ (2.8 Å). It follows that there is no interaction of huprine 19 with Trp82 in the choline-binding pocket nor with Tyr332 in the P-site. However, the electron density revealed the presence of an unidentified linear ligand that occupies the space between Tyr332, Phe329 and huprine 19 (orange sticks in Figure 5D). A very similar ligand was observed in the X-ray structure of the BChE-tacrine complex [24].

#### 2.1.5. Ethopropazine

Ethopropazine is a substituted phenothiazine (Figure 2) with a marked specificity for BChE. The 9000-fold difference in Ki between hAChE and hBChE reflects this specificity [40]. Crystals of the BChE_CHO_-ethopropazine complex were grown by cocrystallization in the presence of 1 mM ligand. The structure was solved at 2.35 Å resolution. The bent shape of the phenothiazine moiety was easily recognizable in the initial electron density map. As already observed for propidium and huprine 19, one aromatic ring is slotted into the groove of the acyl-binding pocket in aromatic T-stacking mode with Trp231 (3.5 Å edge to indole plane distance) and Phe329 (3.9 Å between Phe329-Cε2 and aromatic plane) (Figure 5E). The bent shape is complementary to the molecular surface defined by Thr120, Gln119 and oxyanion hole residues Gly116 and Gly117. Nevertheless, there is sufficient room left for a water molecule to fill the oxyanion hole, stabilized by H-bonds to Ser198-Oγ (2.6 Å) and the main chain nitrogens of Gly116 (2.8 Å) and Gly117 (2.7 Å). Electron density of the protonated alkyl amine substituent is disordered, preventing attribution of the stereochemistry, but two likely conformations of the ethopropazine R-isomer were tentatively modeled to account for the electron density. In one conformation, the diethylamino group points toward Trp82-Cγ (3.5 Å) but without facing the indole ring; in the other conformation, it points toward Tyr332-OH (3.2 Å). The tertiary ammonium does not occupy the choline binding pocket, as seen for the quaternary ammonium of propidium, but gravitates toward the P-site. This particular orientation might be forced by the presence of a heavy ion in the choline binding pocket. Since a chloride anion does not have sufficient electrons to explain the strong rounded density facing Trp82, we arbitrarily chose to model it with a heavier zinc cation that would be eventually stabilized by Glu197 and the electron rich Trp82. However, it could be equally well modeled by a bromide anion of similar electron density, stabilized by the tertiary ammonium substituent of ethopropazine.

### 2.2. Inhibitor Competition

**Figure 6:**
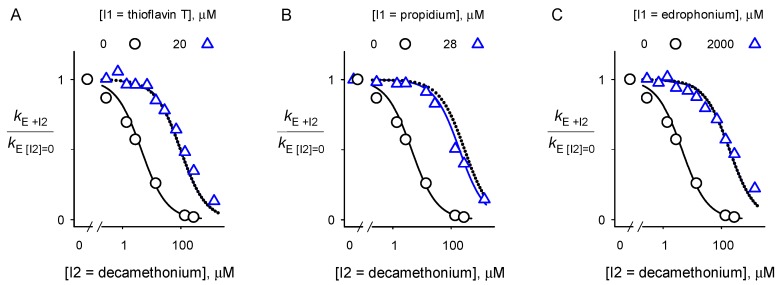
Analyses of relative second order rate constants for substrate hydrolysis by BChE with decamethonium (I2) in the presence (triangle) or absence (circle) of another inhibitor (I1) (**A**–**C**). Values of *k*_E_ were obtained with Equation (1). *K*_2_ for decamethonium was fitted to Equation (5) with [I1] = 0 (solid curves on left of each panel). *K*_1_ for the other inhibitors was obtained in similar fashion from Equation (5) (Table 1). *K*_12_ for decamethonium was fitted to Equation (5) with the [I1] indicated in each panel (solid curves on right of each panel). All experiments were performed in triplicate and the values were averaged. Dotted lines represent the theoretical plot that denotes complete competition between I1 and I2 (i.e., 1/*K*_12_ fixed at 0).

**Figure 7:**
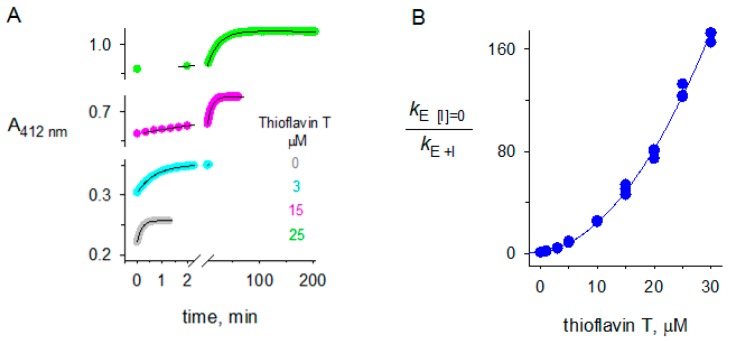
Thioflavin T inhibition of butyrylthiocholine hydrolysis by BChE. (**A**) Examples of experimental traces that were analyzed with Equation (1) to obtain values of *k*_E_. The substrate S was butyrylthiocholine ([S]_0_ = 5 µM), the inhibitor I was thioflavin T (ThT) ([I] as indicated), and [*E*]_tot_ was 0.68 nM; (**B**) Values of *k*_E + I_ obtained from traces like those in Panel A were analyzed with Equation (4) assuming constant percent error in the measured *k*_E[I] = 0_/*k*_E + I_ values to obtain the fitted parameters *K*_I_ = 1.61 ± 0.11 µM and *K*_II_ = 5.84 ± 0.16 µM^2^. These values corresponded to a maximum for the affinity in the binary complex relative to that in the ternary complex (*K*_I12_/*K*_I1_) of 0.56 ± 0.04.

To further compare the binding sites of inhibitors with AChE and BChE, we employed an inhibitor competition assay. This assay is designed to detect any ternary complex formed when two inhibitors are added to AChE or BChE simultaneously [15,23,34,41,42,43]. The assay is conducted with a substrate like acetylthiocholine whose second-order hydrolysis at low substrate concentrations is diffusion controlled and thus is inhibited by ligands that bind to either the A-site or the P-site [15] (see Figure 7A below). Individual inhibition constants *K*_I_ were obtained by plotting the ratios of the hydrolysis rate constant *k*_E_ at various concentrations of one inhibitor (*k*_E + I_) to *k*_E_ in the absence of inhibitor (*k*_E [I] = 0_) against inhibitor concentration [15,43]. Inhibitor competition was then assessed by determining ratios of *k*_E_ at a fixed concentration ([I1]) of inhibitor 1 and various concentrations ([I2]) of inhibitor 2 (*k*_E + I2_) to *k*_E_ at the same [I1] in the absence of [I2] (*k*_E [I2] = 0_) and plotting these ratios against [I2]. These plots were analyzed with Equation (5) (described in the Materials and Methods Section 4.4) to obtain the relative affinity of I1 in its binary complex with enzyme E to I1 in a ternary complex with I2 and E (*K*_12_/*K*_1_). This analysis is shown in Figure 6, where I1 is either ThT, propidium, or edrophonium and I2 is decamethonium.

Each plot with both I1 and I2 is essentially superimposable with the dotted curves that indicate complete competition (i.e., 1/*K*_12_ ≅ 0). The fitted values of the affinities of I1 and I2 in the binary complexes relative to those in the ternary complex (*K*_12_/*K*_1_ = *K*_21_/*K*_2_) are shown in Table 1.

Their large values (i.e., >~100) confirm much higher affinities in the binary complexes, equivalent to complete competition for each inhibitor pair. This is the expected result, as the crystal structure of the BChE-decamethonium complex shows the ligand spanning the A- and P-sites and thus blocking ternary complex formation with either an A-site or P-site inhibitor.

### 2.3. Thioflavin T Inhibition of BChE

The crystal structure of the complex of ThT with BChE showed two ligand molecules in the enzyme active site. It is rare for the active site of an AChE or BChE complex to bind two molecules of the same ligand. Since binding of a ligand to either the A-site or the P-site inhibits second-order hydrolysis of acetylthiocholine, one would predict that two molecules of ThT binding within the active site of BChE would result in plots of *k*_E [I] = 0_/*k*_E + I_ that increase with a second-order dependence on ThT concentration. This dependence is in fact observed in Figure 7B. Comparison of the empirical fitting with Equation (4) in Figure 7B with the mechanistic fit in Equation (3) gave a maximum for the affinity in the binary complex relative to that in the ternary complex (*K*_I12_/*K*_I1_) of 0.56 ± 0.04. This maximum is reached when *K*_I1_ = *K*_I2_. The value 0.56 implies a slightly higher affinity in the ternary complex than in the corresponding binary complex and thus slight positive cooperativity. If *K*_I1_ and *K*_I2_ are not equal, *K*_I12_/*K*_I1_ becomes smaller and implies even more cooperativity. Since little, if any cooperativity, has been observed when different ligands form ternary complexes in the active site of AChE or BChE ([23,43], it is likely that the affinities of ThT for the A- and P-sites in BChE are about equal.

### 2.4. Isothermal Titration Calorimetry

Isothermal titration calorimetry (ITC) measures binding interactions by detecting the heat absorbed or released during a binding reaction. ITC is therefore a universal method and has been applied to a wide range of chemical and biochemical binding interactions [44]. Ligand association with a protein typically involves changes in the intramolecular and intermolecular interactions and dynamics of the system components, including the protein, the ligand, structural and bulk water, and additional components that may be present. The changes in bonding interactions and dynamics that occur upon ligand binding are reflected in the reaction enthalpy and entropy, which, in turn, determine the free energy of ligand association.

In order to complete the characterization of the cholinesterase-ligand interactions, an extensive ITC study was undertaken using recombinant hAChE and hBChE both produced in Chinese hamster ovary (CHO) cells. For most enzyme-ligand pairs, three independent experiments were performed. The measured dissociation constant (*K*_D_), stoichiometry (n), binding heat (ΔH), and Gibbs free energy (ΔG) are summarized in Table 2.

It is important to note that the reported values are based on our best knowledge of both enzyme and ligand concentrations. Thus, errors in the precise active enzyme or ligand concentrations cannot be ruled out. In addition, purity of the ligands, especially the enantiopurity, are potential variables for the determination of exact *K*_D_ values and stoichiometries, due to the likely enantioselectivity of cholinesterases for the different ligands (Reference [45] and structures described therein). However, general trends can be observed from the measured data. The opposite specificities of edrophonium and ethopropazine towards AChE and BChE are clearly observed, with the respective *K*_D_ values presenting about a 20-fold difference. On the contrary, no clear specificity to hAChE or hBChE is observed in the binding of propidium, with only a 2.5-fold difference in *K*_D_ values. For ThT, *K*_D_ is about 7-fold higher for AChE than for BChE. It is noteworthy that these trends observed for *K*_D_ are similar to those of the measured inhibition constants (*K*_I_; see Table 3 below) and values are of the same order (low µmolar range). The stoichiometry data is of greater interest. Values of n are close to 1 for edrophonium, propidium and ethopropazine with both AChE and BChE. A value of n ~ 1 for racemic ethopropazine solution is in agreement with a moderate 2.6-fold enantioselectivity of BChE for the R-isomer in inhibition experiments of acetylthiocholine hydrolysis [45]. However, the n value is close to 2 for ThT with BChE, while with AChE the value of n is close to 1. This difference in n is in accordance with the crystallographic and enzyme inhibition data for ThT reported above.

## 3. Discussion

### 3.1. Is There a True BChE P-Site?

A large number of ligands that bind either to the A-site or the P-site of AChE have been identified, both by X-ray crystallography and by kinetic analysis of inhibitor competition. Fewer ligand complexes with BChE have been reported, but several more are analyzed in this report. Since some aromatic side chains in residues that line the active site gorge in AChE are replaced with aliphatic side chains in the active site of BChE, the P-site in BChE is less well defined. Despite the large volume of the active site gorge available in the region of the BChE P-site, no ligand that binds exclusively to this P-site has yet been identified. Decamethonium spans the P-sites to the choline-binding pockets (A-sites) in both AChE and BChE (Figure 3), as do bis-aromatic benzylpyridinium compounds in BChE [25]. Propidium is a P-site ligand in AChE, but a single bound propidium in BChE extends from the acyl- and choline- binding pockets to the P-site Tyr332 (Figure 5B). ThT is a P-site ligand in AChE, but two molecules of ThT bind in the active site gorge of BChE, and one is in contact with Tyr332 and Trp82 and therefore binds to both A-site and P-site (Figure 4). There is no doubt that the Tyr332-Asp70 duet plays an important role in substrate trafficking and ligand binding, but in the absence of a ligand that binds exclusively in their region at the BChE gorge entrance, and given that a binding site is defined by a ligand, one can legitimately question the existence of a true BChE P-site. At this point, the BChE P-site should be considered as a subsite rather than a full site.

### 3.2. Dual Binding of Thioflavin T to BChE

ThT was reported previously to bind non-competitively with propidium and edrophonium to BChE [23]. A revised analysis of the previous data now gives significantly different values for the inhibition constants with BChE. Values of *K*_I_ for individual inhibitors are lower and values of *K*_12_/*K*_1_ are higher than previously reported, with *K*_12_/*K*_1_ values of 23 and 60 for the ThT-propidium and ThT-edrophonium pairs, respectively (Table 3). These values indicate that ternary complexes of both inhibitors with BChE can form but that the inhibitor affinities in the ternary complexes are much lower than those in the corresponding binary complexes. This conclusion is consistent with the crystallography data, which indicate significant overlap of bound propidium with both molecules of bound ThT.

The complex of BChE with two molecules of bound ThT is also noteworthy because very few ligands complexed with AChE (or BChE) show crystal structures in which two molecules of the same ligand are bound simultaneously. Such structures involving 4-ketoamyltrimethylammonium or choline with AChE [4,5] and acetylcholine with the S203A mutant of AChE [5] involve simultaneous binding of ligand to the A- and P-sites. Recently, AChE complexes with two or more bound ligands have been deduced from kinetic data for hopeahainol A [46] and from both kinetic data and the crystal structure with the dye crystal violet [47]. Neither of these complexes appear to include ligand binding to the A-site, but instead involve simultaneous ligand binding near the entrance of the active site, possibly including the P-site.

### 3.3. Specific Binding of Aromatic Compounds to the BChE Acyl-Binding Pocket

**Figure 8:**
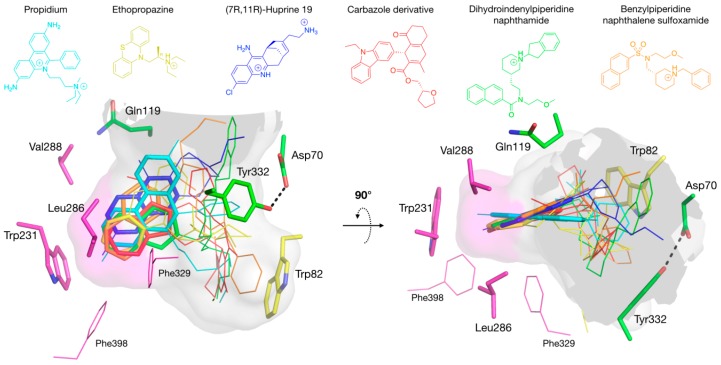
Superimposition of complexes of hBChE with aromatic compounds. In the A-site, choline-binding pocket residues are in yellow and acyl-binding pocket residues in magenta. P-site residues are in green. The gorge is depicted by its molecular surface (semi-transparent gray and magenta for the acyl-binding pocket). The ligands are represented in lines with the aromatic system involved in acyl-binding pocket interaction in sticks: propidium in cyan (pdb 6esj); ethropropazine in yellow (pdb 6eqp); huprine 19 in blue (pdb 6eqq); carbazole derivative in red (pdb 5k5e); dihydroindenylpiperidine naphthamide in green (pdb 4tpk); and benzylpiperidine naphthalene sulfoxamide in orange (pdb 5dyw).

Studies of BChE with a series of N-10-carbonyl derivatives of phenothiazine [48,49,50,51] as well as N-10-alkyl phenothiazines, such as ethopropazine [45], indicate that the location of these bound ligands depends on Tyr332 and Phe329 of the E-helix, whose side chains project into the active site gorge [50]. The structure of the BChE-ethopropazine complex confirmed the importance of these residues, Tyr332 being at cation-π interaction distance of the diethylammonium group, and Phe329 in aromatic T-stacking with the phenothiazine moiety (Figure 5E). In this complex, Phe329 appears to play an important role together with Trp231 in stabilizing an aromatic system in the groove of the acyl-binding pocket. The ability of the acyl-binding pocket in BChE to accommodate aromatic rings without any conformation change stands in contrast to AChE and was already seen in the crystal structure of the o-cresyl-phosphoryl conjugate [52]. In addition to the BChE-ethopropazine complex, the complexes of BChE with ThT, propidium and huprine 19 reported herein and previously published complexes of BChE with carbazole [30], nitroxoline [26] and naphthalene [27,28,29] derivatives demonstrate that the BChE acyl-binding pocket is a major region of interaction for multi-ring aromatic compounds, with their diverse ring-substituents interacting with Tyr332 and/or Trp82 (Figure 8).

This observation markedly differs from the case with AChE, in which a major site of interaction for polycyclic aromatic compounds is the cluster of aromatic residues at the P-site. Actually, the replacement of residues Tyr124, Phe295 and Phe297 in the AChE acyl-binding pocket by the smaller BChE residues Gln119, Leu286 and Val288 provides sufficient room to accommodate these polycyclic ligands. It is also noteworthy that polycyclic aromatic ligands with up to four orders of magnitude of preferential affinity for BChE over AChE [27,40] are likely to bind to the BChE acyl-binding pocket but are unable to bind to the AChE P-site. It follows that targeting the acyl-binding pocket for designing specific inhibitors of BChE is a strategy of choice.

### 3.4. Molecular Docking to the AChE and BChE Active Site Gorges

Molecular docking has become an important tool for the design of AChE inhibitors with improved affinity and selectivity. But docking is quite challenging in the AChE active site because multiple X-ray structures have revealed a large mobility of aromatic P-site and A-site residues to accommodate ligands, especially Trp286 and Tyr337 in hAChE. Therefore, the flexibility of aromatic side chains must be taken into account in docking strategies, either by docking a candidate ligand to various representative conformations of AChE or by allowing side chain flexibility during the docking search as performed by software like Autodock Vina [53]. This complication leads to a considerable increase in calculation time and prevents the screening of large virtual ligand databases. Furthermore, changes in main chain positions are often observed upon ligand binding and cannot be predicted by current docking software. Time-consuming molecular dynamics simulations of the AChE ligand complex is the sole method overcoming this issue, but this approach is unsuitable for large database screening. Another factor that alters docking accuracy in AChE is the consideration of the highly-conserved network of water molecules that significantly contribute to the gorge environment [54] (Figure 1A). Omitting these water molecules during docking could lead to unrealistic binding poses. Consideration of the protonation state of Glu202 and His447 may also have a serious impact on docking pertinence. Indeed, both residues could be either protonated or deprotonated at physiological pH [31], thus affecting the electrostatic field in the A-site and changing an H-bond acceptor to a donor and vice-versa.

In stark contrast to AChE, the crystal structures of BChE in complex with reversible ligands reveal no mobile gorge residues. The largest changes of conformation observed for hBChE occur in the acyl-binding loop Val280-Val288 when the catalytic serine is phosphylated with bulky organophosphates like DFP [2], tabun and derivatives [55], or V-agents [56]. In the absence of mobile residues, molecular docking is facilitated and more reliable. Previous failures to predict the correct binding mode of ethopropazine to BChE are simply the results of docking either to a BChE homology model predating the X-ray structure [40,57] or to a model of an acetylated enzyme [45]. Two recent success stories illustrate the suitability of hBChE for drug discovery by virtual screening of large ligand libraries [27,30]. In both cases, highly selective ligands of BChE with nanomolar affinity were identified. Such a success seems out of reach with AChE, for which subnanomolar ligands are generally discovered by rational design (e.g., huprines [34,36,37]).

## 4. Materials and Methods

### 4.1. Enzymes and Chemicals

Two recombinant hBChE enzymes were used for crystallography. The first (BChE_CHO_), a truncated monomer containing residues 1-529 whose tetramerization domain and four N-glycosylation sites were deleted, was expressed in Chinese hamster ovary (CHO) cells [58]. The second (BChE_S2_) was a truncated monomer containing residues 1-529 expressed in Drosophila S2 cells [38]. Both BChE_CHO_ and BChE_S2_ were secreted into serum-free culture medium and purified by affinity and exclusion chromatography as previously described [38]. BChE_CHO_ crystals are preferred over BChE_S2_ crystals because they are generally of better quality. BChE_S2_ was used as a backup in case of failure to obtain the structure of the complexes with BChE_CHO_.

For enzyme kinetics experiments, purified recombinant hAChE (~1500 units as determined by the supplier) was purchased from Sigma Aldrich (St. Louis, MO, USA). The AChE concentration was calculated using the assumption of 450 units/nmol (4.8 ∆A412/(min × nM)). Purified human plasma wild-type BChE was a gift from Dr. Oksana Lockridge (University of Nebraska Medical Center, Omaha, NE, USA). BChE concentration was calculated assuming 62.5 unit/nmol (0.94 ∆A412/(min × nM)) [59]. As defined previously, 0.1 unit is the amount of BChE that gives 1.0 ∆A/min in the presence of 1.6 × 10^−4^ M butyrylthiocholine in a 1.5 mL assay [60].

For isothermal titration calorimetry, BChE_CHO_ and AChE_CHO_ were used. AChE_CHO_ is a truncated monomer of hAChE containing residues 1-543 (no tetramerization domain) expressed in CHO cells [61].

ThT chloride, decamethonium bromide, ethopropazine hydrochloride, propidium iodide, and edrophonium chloride were purchased from Sigma (Saint-Quentin-Fallavier, France), and huprine 19 was provided by ChemForAse (Rouen, France). ThT was recrystallized, as described previously [12].

### 4.2. Crystals of the hBChE Complexes

#### 4.2.1. Decamethonium and Ethopropazine

BChE_CHO_ was crystallized using the hanging drop vapor-diffusion method as described previously [58]. The mother-liquor contained 1 mM decamethonium or ethopropazine in 0.1 M MES, with 2.1 M ammonium sulfate at pH 6.5. The crystals were grown over a couple of weeks at 20 °C. Crystals were then washed for a few seconds in a cryoprotectant solution (0.1 M MES buffer, 1 mM ligand, 2.3 M ammonium sulfate, 18% glycerol) before being flash-cooled in liquid nitrogen for data collection.

#### 4.2.2. Huprine 19

Crystals of BChE_CHO_ were grown using the hanging drop method in 0.1 M Tris/HCl and 2.3 M ammonium sulfate at pH 8.0. The crystals were then soaked for one hour in the mother liquor containing 1 mM of racemic huprine 19. Crystals were then washed for a few seconds in a cryoprotectant solution (0.1 M Tris buffer, 1 mM huprine 19, 2.3 M ammonium sulfate, 20% glycerol) before being flash-cooled in liquid nitrogen for data collection.

#### 4.2.3. Thioflavin T and Propidium

BChE_S2_ was crystallized using the hanging drop method at a concentration of 5 mg/mL from 0.1 M ammonium acetate buffer, 18% PEG 3350 (pH 7.4) containing either 500 µM of ThT or 1 mM propidium. Crystals were flash-cooled in liquid nitrogen after washing in a cryo-protectant solution composed of the mother liquor supplemented with 20% glycerol prior to data collection.

### 4.3. X-ray Data Collection and Structure of BChE-Ligand Complexes

Diffraction data were collected at the European Synchrotron Radiation Facility (ESRF, Grenoble, France) at different beam lines (ID14-4, ID23-1, ID23-2 and ID29-1). All data sets were processed with XDS [62], intensities of integrated reflections were scaled using XSCALE, and structure factors were calculated using XDSCONV. The structures were solved with the CCP4 suite [63] using the recombinant BChE structure (PDB entry 1P0I and 4AQD) as the starting model. The initial models were refined by iterative cycles of model building with Coot [64], then restrained and subjected to TLS refinement with Phenix [65]. The ligands and their descriptions were built using phenix.elbow included in Phenix. Data collection and refinement statistics are reported in Table S1. Protein structures were illustrated using the program PyMOL 1.8.2 (Schrödinger, Mannheim, Germany). 2Fo-Fc electron density maps of the ligands are represented in Figure S1. The molecular surfaces of the gorges were represented in PyMol with the help of the program Hollow 1.2 [66].

### 4.4. Inhibition of Second-Order Substrate Hydrolysis

Inhibition constants for inhibitors of BChE were determined using a modification of a previously described method [15]. Briefly, varying amounts of inhibitor (in 50 μL of 50% CH_3_CN_(aq)_) were added to 1.60 mL of buffer (0.09 M phosphate buffer, pH 8.0), 5,5′-dithio-bis (2-nitrobenzoic acid) (0.32 mM), and butyrylthiocholine substrate (5 μM) in a quartz cuvette of 1 cm path length, and the mixture was zeroed at 412 nm. The reaction was initiated by the addition of 50 μL of BChE (to 0.68 nM) in 0.1% aqueous gelatin. Assays were conducted at 23 °C. The absorbance (A), reflecting the cumulative hydrolysis of the substrate, was recorded every 5–20 s using an Ultraspec 2100 pro ultra violet (UV)-visible spectrophotometer (Fisher Scientific, Toronto, ON, Canada) equipped with Swift II application software. The fitting of kinetic data with inhibitors was conducted by nonlinear regression with SigmaPlot 12.0 (Systat Software, San Jose, CA, USA), and analyses were either unweighted (Equation (1)) or weighted assuming that the dependent variable had constant percent error [67] (Equations (2)–(5)).

Second order rate constants for substrate hydrolysis (*k*_E_) were obtained from Equation (1) at low initial substrate concentration ([S]_0_) (i.e., [S]_0_ ≤ ~0.2 *K*_app_, [68]) where *K*_app_ is the apparent Michaelis constant) [15] and [S]_0_ is the initial concentration of substrate.

(1) $$A_{412}=A_{412\left(\mathrm{final}\right)}-\Delta A_{412}e^{-k_{obs}t}$$

In Equation (1), A_412_ is absorbance, [E]_tot_ is the total enzyme concentration, *k_obs_* = *k*_E_[E]_tot_, and ΔA_412_ = A_412(final)_ − A_412(t = 0)_.

With substrates that are hydrolyzed by AChE or BChE at nearly diffusion controlled rates, inhibitor binding to either the A- or the P-site, but not both, can be analyzed with Equation (2) [43],

(2) $$\frac{k_{E[I]=0}}{k_{E+I}}=1+\frac{I}{K_{I}}$$

where *k*_E[I] = 0_ is *k*_E_ measured in the absence of inhibitor, *k*_E + I_ is *k*_E_ measured with a fixed concentration of inhibitor I, and *K*_I_ is the inhibition constant (equilibrium dissociation constant) for the enzyme-inhibitor complex. 

When inhibitor binds to two sites simultaneously, and binding to either site can inhibit enzyme activity (for example, both the A- and the P-sites), Equation (3) is used for analysis.

(3) $$\frac{k_{E}{{}_{\left[I\right]}}_{=0}}{k_{E+I}}=1+\frac{\left[I\right]}{K_{I1}}+\frac{\left[I\right]}{K_{I2}}\left(1+\frac{\left[I\right]}{K_{I12}}\right)$$

In Equation (3), *K*_I1_, *K*_I2_, and *K*_I12_ are the equilibrium dissociation constants for I with the A-site, I with the P-site, and I with the A-site in the complex of *E* with I in the P-site, respectively. Equation (3) shows a second-order dependence of *k*_E[I] = 0_/*k*_E + I_ on [I], but the empirical equation for a second-order dependence, given in Equation (4), provides just two fitted parameters, *K*_I_ and *K*_II_.

(4) $$\frac{k_{E}{{}_{\left[I\right]}}_{=0}}{k_{E+I}}=1+\frac{\left[I\right]}{K_{I}}+\frac{{\left[I\right]}^{2}}{K_{\mathrm{II}}}$$

Equating the right-hand sides of Equations (3) and (4) gives *K*_I12_/*K*_I1_ = *K*_II_/(*K*_I_(*K*_I1_ + *K*_I2_)), where *K*_I12_/*K*_I1_ is the affinity of I at the A-site in the binary complex relative to that at the A-site in the ternary complex. *K*_I12_/*K*_I1_ is maximized when *K*_I1_ = *K*_I2_.

### 4.5. Inhibitor Competition

Determining rates of substrate hydrolysis in the presence of two inhibitors provides information that indicates whether these inhibitors are interacting with the same or different enzyme binding sites [15]. To conduct an inhibitor competition assay with AChE or BChE and a diffusion-controlled substrate, a series of assays are carried out with a fixed concentration of one inhibitor I1, here being one of propidium, ThT, or edrophonium, together with varying concentrations of the second inhibitor I2, here being decamethonium. The value of *k*_E_ in the presence of both inhibitors (here denoted *k*_E + I2_) relative to *k*_E_ when only I1 is present (here denoted *k*_E[I2] = 0_) is given by Equation (5) [41].

(5) $$\frac{k_{E+I2}}{k_{E[I2]=0}}=\frac{K_{2}\left(1+\left([I1]/K_{1}\right)\right)}{K_{2}\left(1+\left([I1]/K_{1}\right)\right)+[I2]\left(1+\left([I1]/K_{12}\right)\right)}$$

In this equation, *K*_1_, *K*_2_, and *K*_12_ are the equilibrium dissociation constants for I1 with *E*, I2 with *E*, and I1 with the *E* I2 complex, respectively. Individual inhibition constants (e.g., *K*_2_) could be determined from Equation (5) with [I1] fixed at 0. When both I1 and I2 are present, *K*_12_ will equal *K*_1_ when a ternary complex can form and there is no binding competition, whereas the value of *K*_1_/*K*_12_ will be much greater than one if the ternary complex of *E* with I1 and I2 cannot form. Therefore, this analysis can be used to detect ternary complex formation.

### 4.6. Isothermal Titration Calorimetry (ITC) Measurements

Experiments were performed using BChE_CHO_ and AChE_CHO_. Protein concentrations were measured directly prior to the experiment by UV/Vis spectroscopy using molar extinction coefficients at 280 nm calculated from the primary sequences (100,185 M^−1^·cm^−1^ and 104,195 M^−1^·cm^−1^ for AChE_CHO_ and BChE_CHO_, respectively). Protein and ligand concentrations suitable for the ITC experiments were determined empirically for every run (see experimental protein and ligand concentrations reported in Table S2). Inhibitors and proteins were prepared in the same buffer: 10 mM HEPES with 10 mM NaCl at pH 7.4 for BChE titrations, and 20 mM Tris-HCl with 50 mM NaCl at pH 8.0 for AChE titrations. All solutions were degassed and sterilized with 0.22 µm filters prior to use. Titration experiments were run in a Low Volume Nano ITC (TA Instruments) at 25 °C. The inhibitors were loaded into the 50-µL titration syringe, the protein into the 171-µL cell, and the reference cell was filled with filtered deionized water. For all titration experiments, the stirring speed was set at 250 rpm. In a typical run, 150-s initial and final baselines were recorded, and a first injection of 0.50 μL followed by 19 automated injections of 2.50 μL were made at 300-s intervals. Reference experiments corresponding to inhibitor injection into the respective buffer solution devoid of any enzyme were also performed. Representative traces of the ITC experiments are shown in Figure S2. Injection heats were determined by integration of the peak areas using the NanoAnalyze software v3.7.5 (TA Instruments). Data were analyzed using the independent binding model fitting function included in the software. The best fit provided values of the binding heat (ΔH), the Gibbs free energy change (ΔG), the entropy change (T∆S), the stoichiometry of binding (n) and the dissociation constant (K_D_).

## Figures and Tables

**Table 1:** Inhibition constants (*K*_I_) for four inhibitors with BChE and binding site competition ratios (*K*_12_/*K*_1_) for each inhibitor in the presence of decamethonium. A *K*_12_/*K*_1_ ratio >> 1 denotes competition at the same binding site.

Compound	*K*_I_ (µM)	*K*_12_/*K*_1_
Decamethonium	4.5 ± 0.2	-
Propidium	0.43 ± 0.06 ^a^	118
Thioflavin-T	0.8 ± 0.1 ^a^	>200
Edrophonium	49 ± 3 ^a^	>200

^a^ updated from data in reference [23].

**Table 2:** ITC measurements for cholinesterase-ligand pairs. *K*_D_ is the dissociation constant, n the binding stoichiometry, ΔH the binding heat, ΔG the Gibbs free energy of binding, and T∆S the entropy change, with ΔG = ΔH − T∆S. Where SDs are listed, the reported values are the mean ± SD of three independent experiments.

Enzyme	Compound	*K*_D_ (±SD) (µM)	n (±SD)	ΔH (±SD) (kJ·mol^−1^)	ΔG (±SD) (kJ·mol^−1^)	TΔS (kJ·mol^−1^)	∆S (J·mol^−1^·K^−1^)
**hAChE**	Edrophonium	1.4 ± 0.4	0.8 ± 0.3	−26.4 ± 7.9	−33.6 ± 0.8	−7.2	−24.2
	Ethopropazine	26.7 ± 14.7	1.5 ± 0.3	−10.9 ± 1.1	−26.4 ± 1.6	−15.5	−52.0
	Propidium	6.7 ± 0.7	0.8 ± 0.1	−45.4 ± 5.0	−29.6 ± 0.3	15.8	52.9
	Thioflavin-T	17.7	1	−23.7	−27.2	−3.5	−11.8
**hBChE**	Edrophonium	27.2 ± 2.4	0.9 ± 0.1	−12.6 ± 1.7	−26.1 ± 0.3	−13.5	−45.3
	Ethopropazine	1.1 ± 0.1	1.0 ± 0.1	−43.6 ± 6.4	−34.1 ± 0.2	9.5	31.8
	Propidium	2.1 ± 0.4	0.8 ± 0.1	−43.1 ± 19.1	−31.7 ± 0.4	11.4	38.2
	Thioflavin-T	2.6 ± 0.5	2.2 ± 0.1	−11.4 ± 0.6	−32.0 ± 0.6	−20.6	−69.1

**Table 3:** Inhibition constants (*K*_I_) for hAChE and hBChE; binding site competition ratios (*K*_12_/*K*_1_) for AChE and BChE for each inhibitor in the presence of thioflavin T [23]. A *K*_12_/*K*_1_ ratio of ~1 indicates no competition at binding sites (NC); high ratios (>100) denote competition at the same binding site (C); and intermediate ratios indicate partial competition (PC).

Compound	AChE		BChE	
	*K*_I_ (µM) ^a^	*K*_12_/*K*_1_^a^	*K*_I_ (µM) ^a^	*K*_12_/*K*_1_^a^
Thioflavin T	3.9 ± 0.4	-	0.8 ± 0.1 ^b^	-
Propidium	7.2 ± 0.6	93 (C)	0.43 ± 0.06	23 (PC)
Edrophonium	0.82 ± 0.05	2.9 ± 0.2 (NC) ^a^	49 ± 3	60 (PC)

^a^ Values of *K*_I_ and *K*_12_/*K*_1_ with AChE and BChE have been recalculated from data obtained in reference [23]. The values of *K*_I_ for AChE are somewhat higher than those previously reported (e.g., references [15,43]) because of a higher ionic strength and buffer component differences; ^b^ *K*_I_ for ThT with BChE is an approximation determined at ThT concentrations below 10 μM, as the dependence of *k*_E [I] = 0_/*k*_E + I_ on the ThT concentration is not linear (see Figure 7B).

## Supplementary Materials

Supplementary materials are available online. Figure S1: 2Fo-Fc electron density maps of the ligands; Figure S2: Representatives traces and fits of ITC experiments; Table S1: Data collection and refinement statistics; Table S2: Concentrations of ligand and protein used for ITC titrations.
