# Supplementary material for: Comparison of the Binding of Reversible Inhibitors to Human Butyrylcholinesterase and Acetylcholinesterase: A Crystallographic, Kinetic and Calorimetric Study
Source: Molecules. 2017 Nov 29;22(12):2098. doi: 10.3390/molecules22122098 (PMC6149722; doi:10.3390/molecules22122098)

Supplementary information

Comparison of the Binding of Reversible Inhibitors to Human Butyrylcholinesterase and Acetylcholinesterase: A Crystallographic, Kinetic and Calorimetric Study.

Terrone L. Rosenberry, Xavier Brazzolotto, Ian R. Macdonald, Marielle Wandhammer, Marie Trovaslet-Leroy, Sultan Darvesh and Florian Nachon

Table S1: Data collection and refinement statistics. Calculated using Phenix (Adams *et al* Acta Cryst. D 2010;66:213-221). R-work=Σ |fo - |fc|| / Σ |fo|, fo and fc are observed and calculated structure factors, R-free set uses about thousands randomly chosen reflections. Statistics for the highest-resolution shell are shown in parentheses.

BChE source	CHO-K1	CHO-K1	CHO-K1	Drosophila S2	Drosophila S2
Ligand	Decamethonium	Ethopropazine	Huprine 19	Thioflavine T	Propidium
pdb code	6ep4	6eqp	6eqq	6esy	6esj
Data collection					
X-ray source - beamline	ESRF - ID14-4	ESRF – ID23-2	ESRF ID29-1	ESRF – ID23-1	ESRF – ID14-4
wavelength (Å)	0.934	0.87260	0,979084	0.97242	1.00442
Resolution range (Å) (highest-resolution shell)	42.39 - 2.3 (2.382 - 2.3)	54.89 - 2.349 (2.433 - 2.349)	54.55 - 2.4 (2.486 - 2.4)	39.35 - 2.8 (2.9 - 2.8)	37.5 – 2.98 (3.087 - 2.98)
space group	I422	I 4 2 2	I 4 2 2	P 21 21 21	P 21 21 21
unit cell parameters (Å)	154.9 154.9 132.2 90 90 90	155.2 155.2 135.3 90 90 90	154.3 154.3 134.1 90 90 90	74.27 79.25 228.92 90 90 90	75.0 79.2 228.3 90 90 90
Total reflections	325422 (18524)	226970 (22192)	302500 (28658)	155637 (11402)	96496 (9357)
Unique reflections	35717 (3390)	34489 (3379)	31862 (3154)	33783 (3197)	27721 (2638)
Multiplicity	9.1 (5.5)	6.6 (6.6)	9.5 (9.1)	4.6 (3.4)	3.5 (3.5)
Completeness (%)	99.30 (95.90)	99.50 (98.57)	99.91 (100.00)	98.51 (94.83)	96.74 (93.55)
Mean I/σ (I)	29.63 (4.82)	19.16 (2.26)	17.14 (2.21)	13.09 (1.01)	10.83 (2.01)
Wilson B-factor	41.24	45.19	46.54	75.75	72.68
R-merge	0.05305 (0.3782)	0.07518 (0.8835)	0.09373 (0.922)	0.07808 (1.073)	0.09197 (0.5627)
R-meas	0.05618 (0.4171)	0.08175 (0.96)	0.0991 (0.9772)	0.08845 (1.264)	0.1087 (0.6604)
R-pim	0.01813 (0.1694)	0.03156 (0.3698)	0.03181 (0.3206)	0.04045 (0.6528)	0.05716 (0.3415)
CC1/2	0.999 (0.939)	0.999 (0.805)	0.999 (0.807)	0.998 (0.531)	0.995 (0.728)
CC*	1 (0.984)	1 (0.945)	1 (0.945)	1 (0.833)	0.999 (0.918)
Refinement statistics					
Reflections used	35643 (3390)	34471 (3378)	31856 (3154)	33659 (3192)	27718 (2638)
Reflections for R-free	1999 (190)	1034 (101)	1594 (158)	1635 (146)	970 (93)
R-work	0.1722 (0.2320)	0.1876 (0.2968)	0.1708 (0.2489)	0.2059 (0.3758)	0.2212 (0.3251)
R-free	0.2114 (0.2739)	0.2360 (0.3659)	0.1988 (0.3078)	0.2615 (0.4117)	0.2995 (0.4474)
CC(work)	0.951 (0.788)	0.961 (0.861)	0.961 (0.904)	0.943 (0.664)	0.937 (0.706)
CC(free)	0.930 (0.772)	0.939 (0.805)	0.937 (0.874)	0.930 (0.405)	0.897 (0.466)
Number of non-H atoms	4614	4647	4610	8775	8810
macromolecule	4211	4225	4211	8370	8379
ligands	158	185	190	364	290
solvent	245	237	209	41	141
Protein residues	523	526	526	1070	1053
RMSD (bonds ; Å)	0.004	0.003	0.003	0.007	0.004
RMSD (angles ; deg)	0.72	0.70	0.64	1.12	0.90
Ramachandran favored (%)	96.72	95.80	95.04	94.09	93.33
Ramachandran allowed (%)	2.70	4.20	4.96	5.82	6.67
Ramachandran outliers (%)	0.58	0.00	0.00	0.10	0.00
Rotamer outliers (%)	0.00	0.22	0.00	0.00	0.00
Clashscore	4.28	4.50	4.17	12.75	18.26
Average B-factor (Å^2)	49.83	57.56	60.38	83.89	81.25
macromolecules (Å^2)	48.12	55.87	59.01	81.61	79.95
ligands (Å^2)	90.08	96.14	88.18	138.47	129.07
solvent (Å^2)	53.19	57.45	62.74	64.70	60.40
Number of TLS groups	1	6	6	2	2

Figure S1: Complexes of human BChE with decamethonium (pdb 6ep4), thioflavin T (pdb 6esy), ethopropazine (pdb 6eqp), huprine 19 (pdb 6eqq) and propidium (pdb 6esj). The ligands are represented in balls and sticks with the volumetric representation of the 2Fo-Fc electron density maps of each ligand contoured at 1 sigma level. Key binding site residues in the vicinity of the ligand are represented in stick.

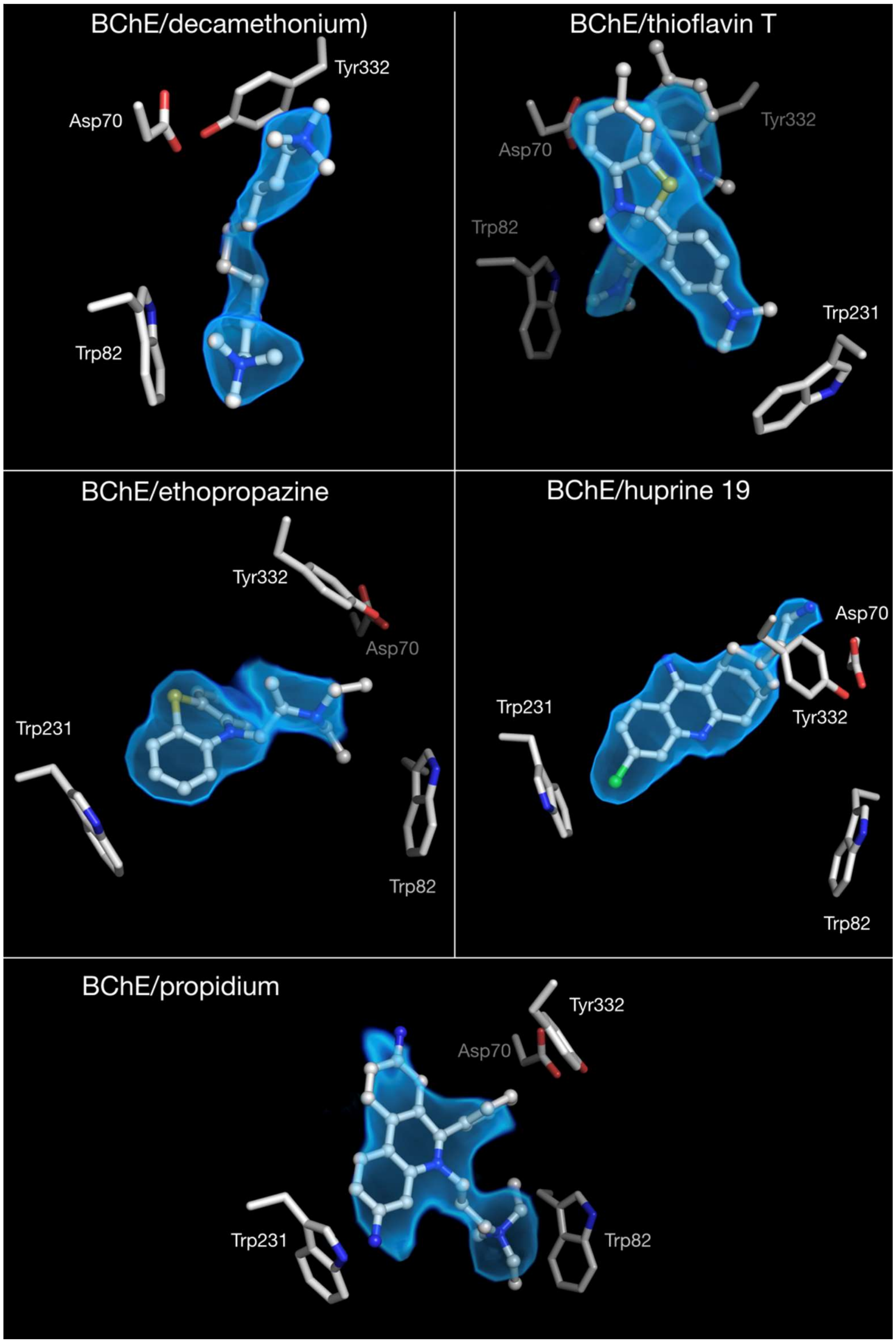

Table S2: Concentrations of ligand and protein used for ITC. Enzyme solutions were prepared from pure stock solutions by dilution into degassed buffer (10 mM HEPES with 10 mM NaCl at pH 7.4 for BChE, and 20 mM Tris-HCl with 50 mM NaCl at pH 8.0 for AChE) and filtered on 0.22μm. Ligand dilutions were prepared from stock solutions prepared in respective buffers (10 mM HEPES with 10 mM NaCl at pH 7.4 for BChE and 20 mM Tris-HCl with 50 mM NaCl at pH 8.0 for AChE) realized by weighing of pure crystalline powder obtained from Sigma, except for ThT chloride which was recrystallized as previously described (Harel *et al* J. Am. Chem. Soc. 2008;130:7856-61).

Compound	Enzyme	[Protein] (μM)	[Ligand] (mM)
Edrophonium	hAChE	55	0.20
		55	0.35
		55	0.50
	hBChE	62	1.00
		133	1.25
		205	5.00
Ethopropazine	hAChE	75	0.75
		75	1.00
		75	1.00
	hBChE	40	0.50
		40	0.25
		40	0.25
Propidium	hAChE	91	0.55
		84	0.88
		75	0.55
	hBChE	80	1.00
		75	0.50
		75	0.30
Thioflavin T	hAChE	53	1.25
	hBChE	30	0.50
		30	0.50

Figure S2: Representative traces and fits of ITC experiments for human AChE (edrophonium and thioflavin T) and human BChE (propidium and thioflavin T). The concentrations of enzyme in the titration cell and concentrations of ligands in the titration syringe are shown. The Injection heats were determined by integration of the peak areas using the NanoAnalyze software v3.7.5 (*TA Instruments*). Data were analyzed using the independent binding model fitting function included in the software. See materials and methods section for further details.

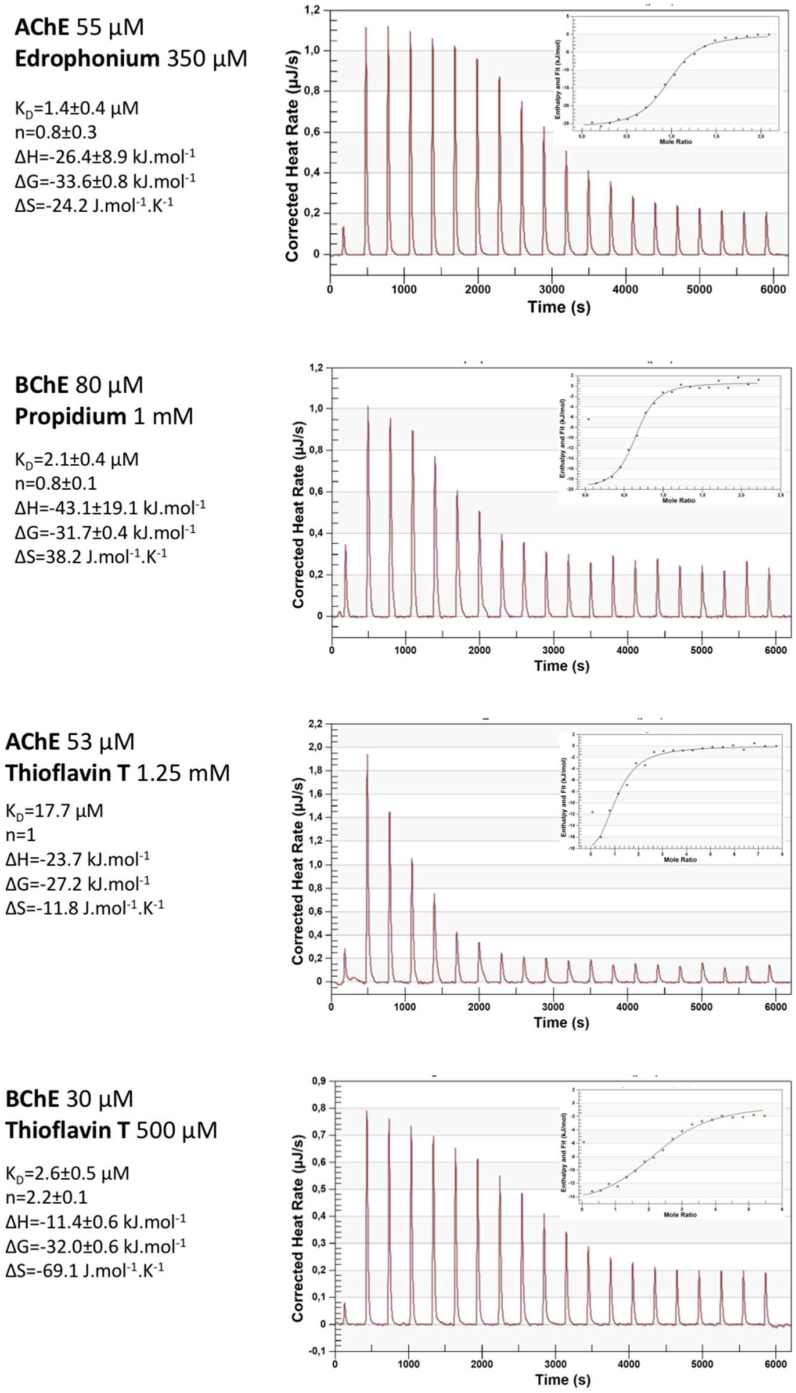

Supplement: Supplementary file 1 [file molecules-22-02098-s001.pdf]
